# The Panel and the Politician

**Published:** 1913-12-13

**Authors:** 


### THE PANEL AND THE POLITICIAN.

With astonishing unanimity, amounting indeed to absolute word-for-word identity, the Ministerial party press has been filling its columns for some days past with fairy tales about the gold-mine which the Insurance Act and the panels are proving to the medical profession. A scare article with appropriate headlines appeared in more than one of these papers, explaining in detail the magnificent prospects which the profession of medicine now offers to its members, the serious shortage of medical men, the rising salaries of assistants, school inspectors and the like, and the thousand a year private practices to be had for the asking under the Insurance Act. When party politicians begin an organised press campaign of this sort, ordinary folk look about for ulterior motives; and in this case they are easy to find.

Beyond question, it is the successful federation of the scattered and feeble non-panel societies into a central and formidable body, the National Medical Union, which has frightened the *Westminster Gazette* and other Radical journals. Hence, the veiled threats of a Royal Commission, and of a State Medical Service.

Already, the incomes of panel doctors are, by implication at least, estimated as excessive in the large crop of articles which has burst out in the daily press. So far, it is merely being argued that newly-qualified men can rely on earning a thousand a year comfortably on the panels; and the notion is held out as a bait to attract more men into joining both the panels and the medical profession itself. Presently, if not already, the extension of the argument will be seen; and an agitation to cut down the pay of the panellers will be based upon the legends now industriously circulated. Thus the exploitation of the medical profession in the supposed interests of politicians and parties, foretold by acute observers as soon as the Insurance Act became law, has actually started; and he will be a bold man who will foretell the end.

But the statements to which we allude are somewhat double-edged. If the panel system does, as alleged, offer a certain, speedy, honourable way of earning a thousand a year to any raw youth who has achieved a medical qualification, there is really no need to grumble about a shortage of doctors; the matter will very quickly right itself in accordance with the doctrine of supply and demand. If, on the other hand, these flaring headlines and sensational articles are merely paving the way for proposals to cut down the capitation fees of those overpaid sinecurists the panellers, all the froth about "Insufficient Doctors," "Doctors' Incomes Trebled," and so forth, is easily explicable.

### Buttressing the British Medical Association.

The truth is, of course, that the National Medical Union has given the Chancellor of the Exchequer a fright, and the measure of his perturbation is reflected in the Ministerial press. Having got the British Medical Association comfortably in his pocket, the Chancellor views with dislike anything which tends to weaken it, or to strengthen a rival professional organisation. The proposals for raising the subscription to the British Medical Association have already caused a good many defections from that body, whose general policy has also disgusted a great many of its members. The view has many times been expressed in these columns that individual defections from the Association, on the part of non-panellers, are futile and unwise; but it is easy to see that the increase of the annual subscription to two guineas will prove just the last straw to many who have contemplated, but not carried out, resignation from the Association. Hence, the fluttering of the party dovecotes, and the remarkable effervescence of party literature to which allusion has been made.

In other words, the medical profession is henceforth to be the football of the party hacks, punted this way or that to serve the electioneering needs of the moment; cajoled, bullied, threatened, flattered by turns to suit the political game.

Questions of an extra shilling or two per head as a capitation fee are insignificant compared with the degradation involved in this ignoble destiny.

### More Experiences from Germany.

Another press device is the printing of information concerning the working of National Insurance in Germany, which is, to say the least of it, unilluminating or even misleading. Thus the *Daily Chronicle* published on December 2 a statement from Berlin that "peace between the doctors and the sick benefit organisations seems to be near." It is true that the evidence adduced in favour of this was flimsy and very scanty; but the statement was the opening sentence of a despatch from "our correspondent," and its optimism may be contrasted with the fact, disclosed a week later, that the Leipzig League (the principal organisation of the German medical profession) has resolved to break off all local negotiations with the *Krankenkassen* throughout Germany, wherever recognition is refused to the League. The Leipzig League, moreover, is making elaborate preparations for a fight; and peace is very far from being as assured as interested parties would like to have it believed. From politicians and party strife the medical profession may well pray to be delivered.

